# 2262: The impact of alcohol dysbiosis on host defense against pneumonia

**DOI:** 10.1017/cts.2017.32

**Published:** 2018-05-10

**Authors:** Derrick Richard Samuelson, Vincent Maffei, Eugene Blanchard, Meng Luo, Christopher Taylor, Judd Shellito, Martin Ronis, Patricia Molina, David Welsh

**Affiliations:** Louisiana State University Health Sciences Center, New Orleans, LA, USA

## Abstract

OBJECTIVES/SPECIFIC AIMS: Alcohol consumption perturbs the normal intestinal microbial communities (alcohol dysbiosis). To begin to investigate the relationship between alcohol-mediated dysbiosis and host defense we developed an alcohol dysbiosis fecal adoptive transfer model, which allows us to isolate the host immune response to a pathogenic challenge at a distal organ (ie, the lung). This model system allowed us to determine whether the host immune responses to *Klebsiella pneumoniae* are altered by ethanol-associated dysbiosis, independent of alcohol use. We hypothesized that alcohol-induced changes in intestinal microbial communities would impair pulmonary host defenses against *K. pneumonia*e. METHODS/STUDY POPULATION: Mice were treated with a cocktail of antibiotics daily for 2 weeks. Microbiota-depleted mice were then recolonized by gavage for 3-days with intestinal microbiota from ethanol-fed or pair-fed animals. Following recolonization groups of mice were sacrificed prior to and 48 hours post respiratory infection with *K. pneumoniae*. We then assessed susceptibility to Klebsiella infection by determining colony counts for pathogen burden in the lungs. We also determined lung and intestinal immunology, intestinal permeability, as well as, liver damage and inflammation. RESULTS/ANTICIPATED RESULTS: We found that increased susceptibility to *K. pneumoniae* is, in part, mediated by the intestinal microbiota, as animals recolonized with an alcohol-induced dysbiotic intestinal microbial community have significantly higher lung burdens of *K. pneumoniae* (5×104 CFU vs. 1×103 CFU) independent of EtOH. We also found that increased susceptibility in alcohol-dysbiosis recolonized animals was associated with a decrease in the recruitment and/or proliferation of CD4+ and CD8+ T-cells (1.5×109 cells vs. 2.5×109 cells) in the lung following Klebsiella infection. However, there were increased numbers of T-cells in the intestinal tract following Klebsiella infection, which may suggest that T cells are being sequestered in the intestinal tract to the detriment of host defense in the lung. Interestingly, mice recolonized with an alcohol-dysbiotic microbiota had increased intestinal permeability as measured by increased levels of serum intestinal fatty acid binding protein (55 vs. 30 ng/mL). Alcohol-dysbiotic microbiota also increased liver steatosis (Oil Red-O staining) and liver inflammation (>2-fold expression of IL-17 and IL-23). DISCUSSION/SIGNIFICANCE OF IMPACT: Our findings suggest that the commensal intestinal microbiota support mucosal host defenses against infectious agents by facilitating normal immune responses to pulmonary pathogens. Our data also suggest that increased intestinal permeability coupled with increased liver inflammation may impair the recruitment/proliferation of immune cells in the respiratory tract following infection. The role of the microbiota during host defense will be important areas of future research directed at understanding the effects of microbial dysbiosis in patients with AUDs.

